# Associations of gender with sexual functioning, loneliness, depression, fatigue and physical function amongst patients suffering from rheumatoid arthritis with a particular focus on methotrexate usage

**DOI:** 10.1007/s00296-024-05555-y

**Published:** 2024-03-14

**Authors:** Laila T. Bay, Dorthe S. Nielsen, Caroline Flurey, Annamaria Giraldi, Sören Möller, Christian Graugaard, Torkell Ellingsen

**Affiliations:** 1 Rheumatology Research Unit, Department of Rheumatology, Odense University Hospital, 5000 Odense, Denmark; 2 Department of Gynecology, University of Southern Denmark, 5000 Odense, Denmark; 3 Department of Geriatrics, Odense University Hospital, Odense, Denmark; 4 Faculty of Health and Applied Sciences, University of the West of England, Bristol, UK; 5 Sexological Clinic, Mental Health Center Copenhagen, Copenhagen University Hospital, Copenhagen, Denmark; 6 Department of Clinical Medicine, University of Copenhagen, Copenhagen, Denmark; 7 OPEN, Odense University Hospital and University of Southern Denmark, Odense, Denmark; 8 Center for Sexology Research, Department of Clinical Medicine, Aalborg University, Aalborg, Denmark

**Keywords:** Rheumatoid Arthritis, Sex, Gender, Methotrexate, Patient-reported outcome measures, cross-sectional analyses

## Abstract

There is a lack of knowledge regarding methotrexate (MTX) usage in patients with rheumatoid arthritis (RA) and its possible links with gender, disease characterization and sexual functioning, loneliness, fatigue and depression. We, therefore, investigated the associations of gender with physical function, fatigue, depression, loneliness and sexual functioning with a particular focus on MTX usage. A cross-sectional study design was used. Inclusion criteria were RA diagnosis, age above 18 years and available data on MTX treatment 1 year after diagnosis. Data consisted of responses from validated questionnaires regarding physical function, fatigue, depression, loneliness and sexual functioning combined with evaluations from medical records. Data were analysed with linear regression models comparing numerical outcome measures between male and female patients and between MTX users and MTX non-users. Amongst 286 patients with RA (69 men and 217 women), 67.8% were MTX users 1 year after diagnosis. Comparing women and men, both overall and within subgroups of MTX usage, we found significantly more adverse outcomes for women than men in physical functioning at diagnosis and in sexual function, depression, fatigue and physical functioning at enrolment in the study. Gender differences were also present when comparing MTX users with MTX non-users divided by gender. There were only significant differences in the HAQ and loneliness scores when comparing MTX users with MTX non-users. Women with RA had more negative outcomes measured by the selected PROMs compared to men with RA, both overall and in subgroups of users and non-users of MTX. These findings call for sharpened attention to the importance of gender in the treatment and care of patients with RA, as well as in future clinical research.

## Introduction

Previous studies on cohorts of rheumatoid arthritis (RA) patients have examined gender differences on parameters such as the course of disease [1], paraclinical markers (RF/anti-CCP status) and other clinical characteristics [2], treatment response [3], symptom burden [3], reporting of symptoms [4] and coping strategies [5].

According to both Danish and international guidelines, methotrexate (MTX) is the first choice of treatment in rheumatoid arthritis (RA) [6], with studies demonstrating a reduced risk of death in patients treated with MTX [7]. Treatment commenced within the first year of diagnosis has proven essential for disease and symptom control [8], targeting low disease activity and eventually remission [9]. Further, one recent study concluded that patient’s belief of illness improved after 1 year of treatment with MTX [10]. However, a large group of RA patients does not respond clinically to MTX or develop gastrointestinal or liver toxicity issues [11]. Despite clear recommendations for MTX use and prescription, dosing of MTX for RA patients seems to be underutilized or suboptimal [12].

There is no agreement in the literature on gender differences in the treatment effect of MTX. While some studies report men responding better to MTX in both early and established RA [13, 14], others report a low response to MTX in women [15]. Such differences have been attributed to hormonal factors [16] or a generally worse global prognosis and increased disability in women with RA [17]. Other studies proved no gender differences concerning MTX response [16, 18].

Depression is one of the most prevalent comorbidity factors amongst patients with RA, and the presence of clinical depression may negatively influence patients’ experience of RA [19], coping ability [19] and treatment adherence [20]. Feelings of loneliness may also be important in an RA trajectory [21]. Loneliness may thus indicate a risk for poorer health, including depression and increased perception of pain [22], and it can negatively affect treatment adherence [23].

Sexual dysfunction can emerge as a consequence of RA symptoms such as fatigue, pain and joint stiffness because of side effects from medical treatment or due to psychological factors such as distress, depression or loneliness [24].

Associations between RA and PROMs, such as depression, loneliness, physical function and sexual functioning, are complex. However, characterizing male and female MTX users and non-users concerning their mental, physical and sexual functioning may improve our understanding of various adverse RA trajectories. Against this background, this study investigates sexual functioning, loneliness, depression, fatigue and physical function in RA patients who, respectively, use and do not use MTX. This may shed light on the emergence of clinically meaningful adversity and vulnerability in specific subgroups of RA patients. Furthermore, the study aims to investigate possible gender differences and gender-specific associations about sexual functioning, loneliness, depression, fatigue and physical function in subgroups of both MTX users and non-users.

## Material and methods

### Study design and patient involvement

The present study was cross-sectional, with consecutive enrolment of patients with RA. As PROMs, the study used selected validated questionnaires (described in detail in the next section) distributed electronically to participants as a joint survey when accepting participation in the study. The electronic survey design was discussed with two patient research partners affiliated with the department of rheumatology, but the questionnaires were not changed.

Data from questionnaires were supplemented with clinical and paraclinical data (described in detail in the next section) collected retrospectively from medical records at each participant’s diagnosis and again after 1 year. Patients were recruited at the outpatient department of rheumatology at a Danish University Hospital, and the electronic survey was distributed to 380 patients between April and September 2018. The collection of data ended in October 2018. Data from the medical records were collected using a structured guide.

Further, the STROBE guidelines were used in the reporting of the study [25].

### Participants

Inclusion criteria included people over 18 years diagnosed with RA (diagnosis codes: M059, seropositive RA; M060, seronegative RA and M069 RA without specification) who could speak and understand Danish without cognitive impairment. Also, inclusion required the availability of medical record data regarding MTX treatment/non-treatment 1 year after diagnosis.

A sample size of 150–200 participants was considered sufficient based on previous cross-sectional studies involving, e.g., the Health Assessment Questionnaire (HAQ) score [26], Becks Depression Index (BDI) [27] and Bristol Rheumatoid Arthritis Fatigue Numeric Rating Scale (BRAF NRS) [28]. All participants signed an informed consent form following both oral and written information and procedures which align with the ethical standards of the Danish Code of Conduct for Research Integrity [29].

### Data collection

#### Retrospective evaluation of medical records

The following data were collected from the electronic patient record at two points in time: (1) treatment with MTX or other synthetic and biologic disease-modifying antirheumatic drug (DMARD) 1 year after diagnosis and (2) seropositivity with IgM-RF- and/or anti-CCP-status; CRP; number of tender joints (NTJ); number of swollen joints (NSJ); Disease Activity Score (DAS)28CRP (based on 28 tender and swollen joints and level of CRP); Charlson’s Comorbidity Index (CCI) and Health Assessment Questionnaire (HAQ) including fatigue (visual analogue scale, VAS), pain (VAS), Patient Global Assessment (PtGA) (VAS) and Physician Global Assessment (PhGA) (VAS) at the time of diagnosis. Disease duration was calculated from the date of diagnosis in the medical record to the date of answering the electronic survey. Data were collected from the electronic medical record, where all clinical and paraclinical data were documented prospectively.

#### Questionnaires included in the electronic survey

##### Patient-related outcomes from the questionnaire

The questionnaire included 11 sociodemographic items: age, gender (male, female and other), cohabitation status (living alone and living together with children and/or other adults), employment status (working and not working), years of education (< or > 12 years of education), daily smoking (cigarettes/cheroots/cigars/pipe), weekly alcohol consumption (units of alcohol/week) (the cut-off values for alcohol consumption being > seven units of alcohol per week (women) and > 14 units of alcohol per week (men), height (m), weight (kg) and body mass index (BMI) (weight(kg)/height(m)^2^) and comorbidity measured by response categories from Charlson´s Comorbidity Index (CCI).

##### Changes in sexual functioning questionnaire (CSFQ-14)

CSFQ-14 is a 14-item gender-specific questionnaire examining changes in sexual functioning due to illness and/or treatment in five domains (desire/frequency, desire/interest, arousal/excitement, orgasm/completion and pleasure). The CSFQ-14 uses a 5-point Likert scale, and the scores range from 14–70, with low scores reflecting a high extent of sexual dysfunction. The cut-off point indicating sexual dysfunction is 47 for men and 41 for women [31]. The CSFQ-14 has been validated in Danish and in a Danish setting [32].

##### The University of California Los Angeles (UCLA) loneliness scale

The UCLA Loneliness Scale is a 20-item questionnaire with responses on a 4-point Likert scale (never, rarely, sometimes or always) [33]. Scoring ranges from 20 to 80 points, with a higher score reflecting higher self-rated loneliness [33]. There is no cut-off point, but some studies have categorized the UCLA scores into four categories: mean score 20–34 (low degree of loneliness), 35–49 (moderate degree of loneliness), 50–64 (moderately high degree of loneliness) and 65 to 80 (high degree of loneliness) [34].

The UCLA Loneliness scale is considered the gold standard for measuring loneliness indirectly and has good psychometric properties [35].

##### Beck’s depression inventory (BDI)

The BDI is a 21-item questionnaire with responses on a 4-point Likert scale (none, mild, moderate and severe) measuring symptoms of depression. Scores range from 0–63, with high scores reflecting a higher extent of depression [36]. Diagnostic categories are ≤ 13 (none or minimal depression), 14–19 (mild depression), 20–28 (moderate depression) and ≥ 29 (severe depression) [36]. The revised version of the BDI was tested on 248 outpatients (Cronbach's alpha = 0.86) [37]. It has been translated into Danish.

##### Bristol rheumatoid arthritis fatigue numerical rating scale (BRAF NRS)

The BRAF NRS contains three items (fatigue, effect on life and coping ability) measured from 0–10 on numeric rating scales: i) fatigue severity describing the average level of fatigue (no fatigue–totally exhausted), ii) effect of fatigue on your life (no effect–a great deal of effect) (items are measured from 0–10, with 0 being no problems) and iii) coping with fatigue (not coping at all well–coping very well) (items are measured from 0–10, with 10 indicating no problems in coping with fatigue) [38]. The BRAF-NRS has been validated in Danish [39].

##### The health assessment questionnaire (HAQ)

The HAQ score is a widely used scoring instrument to measure physical disability in rheumatology. It covers eight domains (dressing, arising, eating, walking, hygiene, reach, grip and daily activities), and it has 20 items with four response options each (without any difficulties, with some difficulties, with many difficulties and unable to do). Scores range between 0 and 3, with a higher mean score indicating increased severity [40]. Three visual analogue scales (VAS) (range: 0–100 mm) on pain, fatigue and the total impact of RA (PtGA VAS) are included in the HAQ instrument but not part of the eight domains and, therefore, not included in the score calculation. The HAQ has been validated repeatedly [41], including adaptation in Danish and a Danish setting [42].

### Statistical methods

A prespecified statistical analysis plan (SAP) was established with a statistician. Linear regression models were applied to compare the numerical outcome measures (HAQ, BRAF-NRS, BDI, UCLA Loneliness Scale and CSFQ) between male and female patients as well as between MTX users and MTX non-users, and in a combined model including an interaction between gender and MTX use. Hence, all our analyses were performed on the whole cohort, considering the desired comparisons by specifying proper interaction structures between gender and MTX status. Furthermore, logistic regression was applied for current smoking and alcohol consumption as outcome measures comparing MTX users and MTX non-users. Unadjusted analyses were conducted, as well as analyses adjusted for patient age, gender, years of education, employment and cohabitation status.

Due to non-normally distributed residuals in the linear regressions (evaluated by quantile–quantile plot and Shapiro–Wilks test resulting in *p*-values < 0.001 for most models), bootstrapping with 1,000 repetitions was applied to estimate confidence intervals and *p*-values. Interaction terms between gender and MTX usage were used to investigate possible gender differences in the secondary outcomes.

The main analysis was conducted on available data to manage missing data. The analyses were repeated for smoking and alcohol consumption by ordinal logistic regression considering the amount of smoking/alcohol consumption. Furthermore, we repeated the analyses on measured pain, fatigue and PtGA scales and for CCI at enrolment rather than at the time of diagnosis.

All statistical analyses were performed using STATA.

## Results

**Fig. 1:**
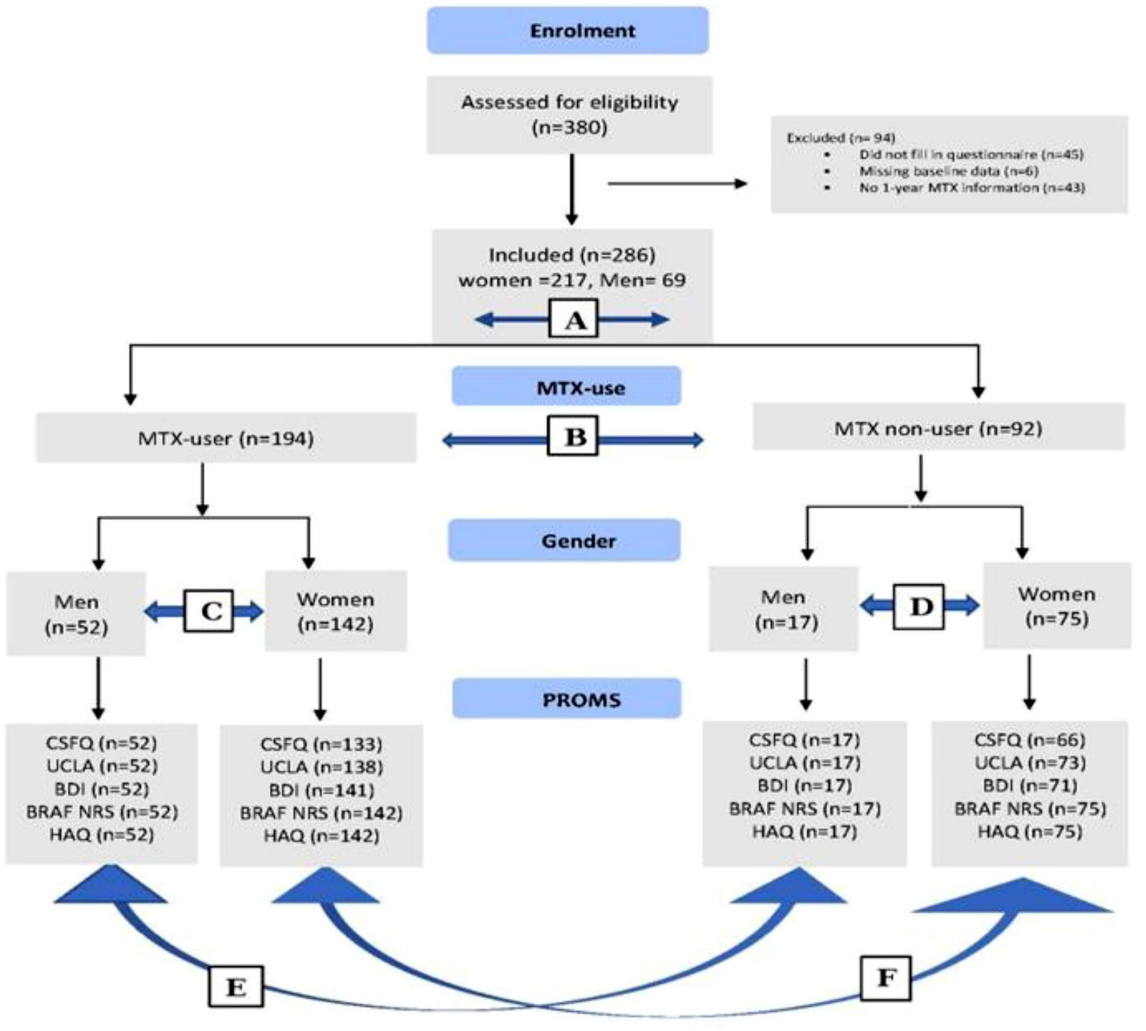
Flowchart of inclusion and analyses. Vertical lines show patient flow. Horizontal lines illustrate groups for comparison by statistical analysis: **A** Women compared to men in both groups/in general **B** MTX users compared with MTX non-users **C** Female MTX non-users compared to male MTX non-users** D** Female MTX users compared to male MTX users **E** Male MTX users compared to male MTX non-users **F** Female MTX users compared to female MTX non-users

Of the 380 participants invited, 286 patients (69 men and 217 women) were included in the analyses (Fig. 1). Participants’ mean age was 45.0 (SD 10.8) years at the time of diagnosis and 56.6 (SD 10.0) years at the completion of the survey. The mean disease duration was 12.1 (SD 8.3) years. Most participants had received more than 12 years of school education (73.0%). Half of the respondents were currently unemployed (50.1%), and one-fourth lived alone (24.1%) (Table 1).

**Table 1:** Clinical and demographic characteristics and patient-reported outcome measures

Demographic and lifestyle factors	All (A)	MTX users (*n* = 194) (C)		MTX non-users (*n* = 92) (D)	
	286 (69 men)	Men, *n* = 52	Women, *n* = 142	Men, *n* = 17	Women, *n* = 75
Age at diagnosis, years, mean [SD]	45.0 [10.8]	48.0 [8.4]	46.7 [10.2]	45.0 [11.1]	39.8 [11.6]
Age at study enrolment, years, mean [SD]	56.6 [10.0]	57.1 [7.9]	55.9 [10.1]	57.6 [10.7]	57.5 [10.9]
Disease duration*, years, mean [SD]	12.1 [8.3]	9.5 [5.7]	9.8 [6.0]	13.0 [7.5]	18.2 [10.4]
> 12 years of education, *n* (%)	209 (73.0)	38 (73.1)	108 (76.1)	11 (64.7)	52 (69.3)
Unemployed, *n* (%)	144 (50.3)	22 (42.3)	65 (45.8)	8 (47.1)	49 (65.3)
Living alone, *n* (%)	69 (24.1)	12 (23.1)	33 (23.2)	2 (11.8)	22 (29.3)
BMI, kg/m2, mean [SD]	26.9 [5.8]	26.8 [4.0]	27.5 [6.9]	28.1 [3.6]	25.8 [4.6]
Smoking (current), *n* (%)	127 (44.4)	24 (46.0)	58 (40.8)	10 (58.8)	35 (46.7)
Alcohol units, > 7/14 units pr. week, *n* (%)	86 (30.1)	21 (40.4)	38 (26.8)	9 (52.9)	18 (24.0)
Disease-specific factors at diagnosis					
Seropositive**, *n* (%)	232 (81.1)	43 (82.7)	112 (78.9)	13 (76.5)	64 (85.3)
Charlson´s Comorbidity Index 0, *n* (%)	171 (59.8)	34 (65.4)	92 (64.8)	11 (64.7)	34 (45.3)
Charlson´s Comorbidity Index 1, *n* (%)	25 (8.7)	3 (5.8)	15 (10.6)	0 (0.0)	7 (9.3)
Charlson´s Comorbidity Index 2+ , *n* (%)	17 (5.9)	4 (7.7)	10 (7.0)	0 (0.0)	3 (4.0)
Disease activity at diagnosis					
CRP*** (mg/L), mean [SD]	19.6 [30.8]	20.1 [28.2]	19.6 [33.1]	14.2 [11.2]	21.6 [32.4]
HAQ (0–3), mean [SD]	1.01 [0.7]	0.7 [0.5]	1.0 [0.7]	0.4 [0.3]	1.6 [0.6)
Number of tender joints (0–44), mean [SD]	8.5 [6.8]	6.8 [5.4]	9.6 [7.0]	4.2 [3.9]	8.2 [7.6]
Number of swollen joints (0–44), mean [SD]	5.5 [4.7]	5.3 [5.2]	5.4 [4.0]	3.7 [3.4]	6.7 [6.5]
DAS28CRP (2–10), mean [SD]	4.5 [1.2]	4.3 [1.2]	4.6 [1.2]	3 .7 [0.0]	4.7 [1.6]
Physician Global Assessment (0–100) VAS, mean [SD]	35.4 [20.3]	31.1 [21.3]	35.4 [18.2]	36.0 [21.4]	41.9 [25.6]
Fatigue (0–100) VAS, mean [SD]	54.0 [27.4]	46.3[26.3]	56.3 [28.2]	45.0 [26.8]	60.9 [24.2]
Pain (0–100) VAS, mean [SD]	50.9 [24.2]	47.0 [25.6]	52.0 [23.1]	43.2 [24.6]	55.7 [26.8]
Patient Global Assessment (0–100) VAS, mean [SD]	55.9 [25.9]	47.5 (22.4]	58.8 [25.8]	38.8 [25.3]	63.1 [28.8]
Medication (exclusive MTX)					
sDMARDs 1 year from diagnosis, *n* (%)	93 (30.5)	18 (34.6)	38 (26.8)	8 (47.1)	29 (38.7)
bDMARDs 1 year from diagnosis, *n* (%)	80 (28.0)	14 (26.9)	34 (23.9)	4 (23.5)	28 (37.3)
Disease activity at enrolment					
HAQ (0–3), mean [SD]	0.8 [0.7]	0.4 [0.6]	0.8 [0.7	0.6 [0.7]	1.1 [0.8]
Fatigue (0–100) VAS, mean [SD]	48.9 [26.1]	41.3 [23.9]	52.5 [26.2]	42.0 [28.8]	48.7 [25.9]
Pain (0–100) VAS, mean [SD]	37.8 [23.6]	31.6 [22.3]	40.3 [24.4]	35.5 [21.5]	37.8 [23.1]
Patient Global Assessment (0–100) VAS, mean [SD]	43.6 [25.6]	36.7 [23.7]	46.1 [26.9]	37.5 [23.1]	44.8 [24.0]
Psychosocial factors at enrolment					
Sexuality					
CSFQ total score (14–70), mean [SD]	39.8 [12.0]	49.2 [9.7]	36.34[10.5]	46.7 [12.4]	37.4 [11.8]
Sexual dysfunction (≤ 47 women, ≤ 41 men), *n* (%)	141 (52.6)	19 (36.5)	80 (60.2)	4 (23.5)	38 (57.6)
Loneliness					
UCLA Loneliness score (20–80), mean [SD]	37.1 [11.2]	36.2 [11.3]	39.0 [11.4]	32.8 [9.6]	35.0 [10.7]
High degree of loneliness (≥ 65), *n* (%)	2 (1.0)	0 (0.0)	2 (1.5)	0 (0.0)	0 (0.0)
Depression					
BDI total (0–63), mean [SD]	9.1 (7.7)	7 .1 [5.9]	10.5 [8.2]	5.6 [3.5]	8 .9 [7.9]
Severe depression (≥ 29), *n* (%)	7 (2.5)	0 (0.0)	5 (3.6)	0 (0.0)	2 (2.3)
Fatigue					
BRAF Numeric Rating Scale Severity (0–10), mean [SD]	5.2 [2.5]	4.4 [2.5]	5.6 [2.5]	4.0 [2.6]	5.2 [2.5]
BRAF Numeric Rating Scale Effect (0–10), mean [SD]	4.9 [2.7]	4.1 [2.7]	5.2 [2.7]	3.7 [2.3]	5.1 [2.7]
BRAF Numeric Rating Scale Coping (0–10), mean [SD]	4.1 [2.6]	3.5 [2.7]	4.4 [2.6]	4.1 [2.70	3.9 [2.6]

*SD* standard deviation, *BMI* body mass index, *CRP* C-reactive protein, *HAQ* health assessment questionnaire, *DAS* disease activity score, VAS visual analogue scale, sDMARD synthetic disease-modifying antirheumatic drug, bDMARD biologic disease-modifying antirheumatic drug, *UCLA* University of California Los Angeles, *CSFQ* changes in sexual functioning questionnaire, *BDI* becks depression inventory, *BRAF-NRS* bristol rheumatoid arthritis fatigue numeric rating scale

^*^The disease duration mentioned in Table 1 is the duration from the time of diagnosis to enrolment in the PROM-based survey

^**^Patients positive for anti-cyclic citrullinated protein or rheuma factor were considered positive

^***^CRP Local reference: < 6 mg/L. A, C and D refer to Fig. 1

The demographic characteristics and disease activity measures are listed in Table 1. The majority of participants (67.8%) were MTX users (n = 194; 52 men and 142 women), while 92 participants (17 men and 75 women) were MTX non-users (Table 1).

### Correlation between PROMs

As shown in Table 2, several PROMs were correlated. The HAQ total score collected in the survey correlated with VAS fatigue, VAS pain, VAS global, the HAQ collected at the time of diagnosis and the Beck Depression Inventory (BDI).

**Table 2:** Spearman’s correlation coefficients between patient-reported outcome measures (PROMs) selected at diagnosis and enrolment in the study

	HAQ score*	VAS fatigue**	VAS pain**	VAS global**	BDI total*	UCLA total*	CSFQ total*	BRAF global*	BRAF effect*	BRAF coping*	HAQ score**	VAS fatigue*	VAS pain*	VAS global*
HAQ score*	1.0000													
VAS fatigue**	*r* = 0.4798 *p* ≤ *0.0001*	1.0000												
VAS pain**	*r* = 0.3066 *p* = *0.0025*	***r*** = **0.6446** *p* ≤ *0.0001*	1.0000											
VAS global**	*r* = 0.4328 *p* ≤ *0.0001*	***r*** = **0.7015** *p* ≤ *0.0001*	***r*** = **0.7055** *p* ≤ *0.0001*	1.0000										
BDI total*	***r*** = **0.5382** *p* ≤ *0.0001*	*r* = 0.2243 *p* = *0.0289*	*r* = 0.0311 *p* = 0.7647	*r* = 0.1447 *p* = 0.1617	1.0000									
UCLA total*	*r* = 0.2146 *p* = *0.0368*	*r* = 0.1099 *p* = 0.2891	*r* = −0.0230 *p* = 0.8249	*r* = −0.0153 *p* = 0.8833	***r*** = **0.5137** *p* ≤ *0.0001*	1.0000								
CSFQ total*	*r* = −0.4256 *p* ≤ *0.0001*	*r* = −0.2021 *p* = *0.0495*	*r* = −0.1288 *p* = 0.2135	*r* = −0.1872 *p* = 0.069	*r* = −0.2756 *p* = *0.0069*	*r* = −0.1942 *p* = 0.0594	1.0000							
BRAF global*	*r* = 0.4817 *p* ≤ *0.0001*	*r* = 0.3127 *p* = *0.0020*	*r* = 0.1196 *p* = 0.2482	*r* = 0.3486 *p* = *0.0005*	***r*** = **0.5302** *p* ≤ *0.0001*	*r* = 0.2425 *p* = *0.0179*	*r* = −0.1841 *p* = 0.0742	1.0000						
BRAF effect*	*r* = 0.4692 *p* ≤ *0.0001*	*r* = 0.2938 *p* = *0.0039*	*r* = 0.1170 *p* = 0.2589	*r* = 0.3193 *p* = *0.0016*	***r*** = **0.5994** *p* ≤ *0.0001*	*r* = 0.2824 *p* = *0.0056*	*r* = −0.1730 *p* = 0.0936	***r*** = **0.9188** *p* ≤ *0.0001*	1.0000					
BRAF coping*	*r* = 0.4677 *p* ≤ *0.0001*	*r* = 0.2370 *p* = *0.0208*	*r* = 0.1717 *p* = 0.0961	*r* = 0.2811 *p* = *0.0058*	***r*** = **0.5779** *p* ≤ *0.0001*	*r* = 0.4600 *p* ≤ *0.0001*	*r* = −0.1436 *p* = 0.1649	***r*** = **0.7035** *p* ≤ *0.0001*	***r*** = **0.7624** *p* ≤ *0.0001*	1.0000				
HAQ score**	***r*** = **0.5149** *p* ≤ *0.0001*	***r*** = **0.5957** *p* ≤ *0.0001*	***r*** = **0.5596** *p* ≤ *0.0001*	***r*** = **0.5784** *p* ≤ *0.0001*	*r* = 0.1325 *p* = 0.2006	*r* = 0.0000 *p* = 0.9996	*r* = −0.3017 *p* = *0.0030*	*r* = 0.2487 *p* = *0.0151*	*r* = 0.2011 *p* = 0.0507	*r* = 0.2261 *p* = *0.0276*	1.0000			
VAS fatigue*	***r*** = **0.5299** *p* ≤ *0.0001*	*r* = 0.3598 *p* = *0.0003*	*r* = 0.1519 *p* = 0.1417	*r* = 0.3586 *p* = *0.0004*	***r*** = **0.5763** *p* ≤ *0.0001*	*r* = 0.2329 *p* = *0.0231*	***r*** = −0.2108 *p* = *0.0403*	***r*** = **0.9034** *p* ≤ *0.0001*	***r*** = **0.8372** *p* ≤ *0.0001*	***r*** = **0.6441** *p* ≤ *0.0001*	*r* = 0.2714 *p* = *0.0078*	1.0000		
VAS pain*	***r*** = **0.5997** *p* ≤ *0.0001*	*r* = 0.3188 *p* = *0.0016*	*r* = 0.1861 *p* = 0.0709	*r* = 0.2939 *p* = *0.0038*	*r* = 0.4548 *p* ≤ *0.0001*	*r* = 0.0959 *p* = 0.3550	*r* = −0.2956 *p* = *0.0036*	***r*** = **0.5619** *p* ≤ *0.0001*	***r*** = **0.5853** *p* ≤ *0.0001*	*r* = 0.4394 *p* ≤ *0.0001*	*r* = 0.3112 *p* = *0.0021*	***r*** = **0.6688** *p* ≤ *0.0001*	1.0000	
VAS global*	***r*** = **0.6631** *p* ≤ *0.0001*	*r* = 0.3467 *p* = *0.0006*	*r* = 0.2001 *p* = 0.0519	*r* = 0.3819 *p* = *0.0001*	***r*** = **0.6072** *p* ≤ *0.0001*	*r* = 0.2071 *p* = *0.0440*	*r* = −0.2857 *p* = *0.0050*	***r*** = **0.6833** *p* ≤ *0.0001*	***r*** = **0.6895** *p* ≤ *0.0001*	***r*** = **0.5221** *p* ≤ *0.0001*	*r* = 0.3389 *p* = *0.0008*	***r*** = **0.7749** *p* ≤ *0.0001*	***r*** = **0.8796** *p* ≤ *0.0001*	1.000

HAQ health assessment questionnaire, VAS visual analogue scale, BDI becks depression inventory, UCLA University of California Los Angeles, CSFQ changes in sexual functioning questionnaire, BRAF bristol rheumatoid arthritis fatigue

*data collected from questionnaires sent to patients

**medical records, data collected at the time of diagnosis in medical records

BDI correlated with all BRAF measures, the total UCLA score, VAS fatigue and VAS global.

The CSFQ did not correlate with other measures, and the UCLA Loneliness score only correlated with the BDI as mentioned above.

### Comparing women and men (overall)

Overall, analyses adjusted for age, years of education, employment and cohabitation status found significant mean differences in several categories between women and men (Table 3, column A): women had a significantly higher mean score than men in the HAQ score at diagnosis (mean difference 0.4; CI 0.2–0.7) and in several other parameters at the time of enrolment in the study: HAQ score (mean difference 0.4; CI 0.2–0.5), fatigue score (VAS) (mean difference 8.8; CI 2.4–15.3), PtGA (VAS) (mean difference 7.4; CI 0.8–14.0), BDI (mean difference 2.8; CI 1.0–4.5), BRAF NRS severity (mean difference 1.0; CI 0.3–1.6) and BRAF NRS effect (mean difference 1.1; CI 0.4–1.7). Further, women had a significantly lower mean CSFQ score than that of men (mean difference -12.2; CI -14.8- -9.5) and a higher percentage of women than men in the MTX user group (60.2% vs. 36.5%) and in the MTX non-user group (57.2% vs. 23.5%) had a mean score indicating a sexual dysfunction (Table 1).

**Table 3:** Adjusted differences between women and men, MTX users and MTX non-users, female and male MTX users, male MTX users, and male MTX non-users, and female MTX users and female MTX non-users

Adjusted analyses*	Comparing women and men (overall) (A)		Comparing MTX users and MTX non-users (overall) (B)*		Comparing women and men (MTX user) (C)		Comparing women and men (MTX non-users) (D)		Comparing male MTX users and male MTX non-users (E)		Comparing female MTX users and female MTX non-users (F)	
	Coefficient (95% CI)	*p*-value	Coefficient (95% CI)	*p*-value	Coefficient (95% CI)	*p*-value	Coefficient (95% CI)	*p*-value	Coefficient (95% CI)	*p*-value	Coefficient (95% CI)	*p*-value
Physical functioning at diagnosis												
HAQ	0.4 (0.2—0.7)	** < 0.001**	-0.3 (−0.6;—0.0)	**0.024**	0.3 (0.0—0.6)	**0.025**	1.1 (0.7—1.5)	** < 0.001**	0.3 (−0.1—0.6)	0.205	−0.5 (−0.8—−0.2)	** < 0.001**
VAS fatigue	7.7 (−4.4—19.7)	0.212	−6.6 (−18.8—5.6)	0.288	6.5 (−6.9—20.0)	0.340	14.6 (−13.7—42.8)	0.313	−0.9 (−28.4—26.6)	0.949	−8.9 (−22.6—4.7)	0.201
VAS pain	4.8 (−5.7—15.2)	0.369	−2.7 (−14.2—8.9)	0.654	4.6 (−6.4—15.7)	0.412	5.6 (−21.7—33.0)	0.686	−1.9 (−26.1—22.3)	0.877	−2.9 (−17.2—11.4)	0.691
VAS PtGA	10.9 (−0.2—22.0)	0.054	−0.9 (−13.1—11.8)	0.893	9.8 (−1.7—21.3)	0.096	18.8 (−11.1—48.7)	0.219	6.2 (−20.2—32.6)	0.645	−2.8 (−17.7—12.1)	0.713
Physical functioning at enrolment in the study												
HAQ	0.4 (0.2—0.6)	** < 0.001**	−0.1 (−0.3—0.0)	0.101	0.4 (0.2—0.6)	** < 0.001**	0.3 (−0.1—0.7)	0.112	−0.2 (−0.6—0.2)	0.274	−0.1 (−0.3—0.1)	0.216
VAS fatigue	8.8 (2.4—15.3)	**0.007**	3.4 (−3.1—9.9)	0.307	11.2 (4.0—18.4)	**0.002**	3.3 (−11.0—18.6)	0.672	−2.8 (−17.9—12.3)	0.716	5.2 (−2.3—12.4)	0.177
VAS pain	5.8 (−0.5—12.1)	0.069	2.5 (−3.4—8.4)	0.408	8.7 (1.4—16.0)	**0.019**	−1.9 (−14.4—10.6)	0.767	−5.9 (−18.6—6.8)	0.363	4.7 (−1.9—11.4)	0.166
VAS PtGA	7.4 (0.8—14.0)	**0.028**	2.4 (−4.0—8.7)	0.464	9.2 (1.5—17.0)	**0.020**	2.8 (−10.8—16.4)	0.688	−2.7 (−16.6—11.2)	0.702	3.7 (−3.5—10.9)	0.312
Psychosocial factors at enrolment in the study												
Sexual dysfunction												
CSFQ	−12.2 (−14.8—−9.5)	** < 0.001**	−1.2 (−3.8—1.4)	0.374	−13.3 (−16.3—10.3)	** < 0.001**	−9.2 (−14.9—−3.5)	**0.002**	2.0 (−3.5—7.4)	0.473	−2.1 (−5.2–0.9)	0.177
Loneliness												
UCLA	1.6 (−1.4—4.5)	0.288	4.2 (1.5—6.9)	**0.003**	2.5 (−0.9—6.0)	0.153	0.4 (−4.6—5.6)	0.885	2.5 (−2.7—7.6)	0.347	4.6 (1.5—7.7)	**0.004**
Depression												
BDI	2.8 (1.0—4.5)	**0.002**	1.8 (−0.01—3.6)	0.051	3.2 (1.1—5.3)	**0.003**	2.1 (−0.5—4.8)	0.112	0.9 (−1.6—3.5)	0.454	2.0 (−0.2—4.2)	0.068
Fatigue												
Bristol Rheumatoid Arthritis Fatigue Numeric Rating Scale												
Severity	01.0 (0.3—1.6)	**0.004**	0.4 (−0.2—1.0)	0.187	1.1 (0.3—1.8)	**0.006**	0.9 (−0.5—2.3)	0.212	0.3 (−1.1—1.7)	0.713	0.4 (−0.2—1.1)	0.201
Effect	1.1 (0.4—1.7)	**0.002**	0.3 (−0.4—0.9)	0.442	1.1 (0.3—1.9)	**0.008**	1.1 (−0.2—2.4)	0.108	0.3 (−1.1—1.6)	0.694	0.2 (−0.5–0.9)	0.521
Coping	0.6 (−0.1—1.3)	0.087	0.3 (−0.3 – 1.0)	0.329	0.9 (0.1—1.7)	**0.022**	−0.3 (−1.8—1.3)	0.749	−0.6 (−2.2—0.9)	0.457	0.6 (−0.2–1.3)	0.120
	Odds ratio (95% CI)	*p*-value	Odds ratio (95% CI)	*p*-value	Odds ratio (95% CI)	*p*-value	Odds ratio (95% CI)	*p*-value	Odds ratio (95% CI)	*p*-value	Odds ratio (95% CI)	*p*-value
Lifestyle at enrolment												
Smoking (yes/no)	0.6 (0.3–0.9)	**0.035**	1.2 (0.8–2.0)	0.381	0.5 (0.3—0.9)	**0.035**	0.8 (0.3—2.1)	0.606	1.68 (0.61–4.62)	0.312	1.1 (0.7—1.9)	0.643
Alcohol (> 7/14 units per week)	0.5 (0.3–0.8)	**0.006**	0.9 (0.5—1.5)	0.588	0.4 (0.2—0.8)	**0.006**	0.6 (0.2—1.7)	0.356	1.17 (0.41–3.30)	0.766	0.8 (0.4–1.4)	0.414

*MTX* Methotrexate, HAQ health assessment questionnaire, VAS visual analogue scale, PtGA patient global assessment, UCLA University of California Los Angeles, CSFQ changes in sexual functioning questionnaire, *BDI* becks depression inventory, *CI* confidence interval

*Column B is adjusted for gender, patient age, years of education, employment and living alone; all other columns are not adjusted for gender

A, B, C, D, E and F refer to Fig. 1

### Comparing MTX users and MTX non-users (overall)

The analysis adjusted for gender, age, years of education and cohabitation status revealed a significantly higher score on the UCLA Loneliness Scale amongst MTX users than amongst MTX non-users (mean difference 4.15; CI 1.5–6.9). No other significant differences were found between these two groups (*p*-values between 0.051 and 0.652) (See Table 3, column B).

### Comparing women and men (MTX users)

Comparing the two MTX user groups (Table 3, column C), stratified by gender, we observed differences between women and men on several parameters: in women, we found an increased mean HAQ score (mean difference 0.4; CI 0.2–0.6), increased mean fatigue score (VAS) (mean difference 11.2; CI 4.0–18.4), increased mean pain score (VAS) (mean difference 8.7; CI 1.4–16.0), increased mean PtGA (VAS) (mean difference 9.2; CI 1.5–17.0), increased mean BRAF-NRS severity (mean difference 1.1; CI 0.3–1.8), increased mean BRAF-NRS effect (mean difference 1.1; CI 0.3–1.9), increased mean BRAF-NRS Coping (mean difference 0.9; CI 0.1–1.7) and decreased mean CSFQ score (mean difference -13.3; CI -16.3- -10.3) compared to men. Furthermore, the risk of smoking and consuming more alcohol than recommended by the Danish Health Authority was half for women in comparison with men (OR 0.5; CI 0.3- 0.9).

### Comparing women and men (MTX non-users)

When comparing female MTX non-users with male MTX non-users (Table 3, column D), there was a significant difference in the CSFQ mean score, indicating increased sexual dysfunction in women (mean difference -9.2; CI -15.0—-3.5) compared to men. No other differences were statistically significant.

### Comparing male MTX users with male MTX non-users and female MTX users with female MTX non-users

Comparing male MTX users with male MTX non-users and female MTX users with female MTX non-users (Table 1), no differences in mean age (57.1 vs. 57.6 and 55.9 vs. 57.5), the proportion of seropositivity (82.7 vs. 76.5 and 78.9 vs. 85.3) or mean DAS-28CRP (4.3 vs. 3.7 and 4.6 vs 4.7) were observed.

Both male and female MTX users had lower mean disease duration than that of male and female MTX non-users, respectively (9.5 vs. 12.9 and 9.8 vs. 18.2), and there was a difference between the HAQ mean score at the time of diagnosis in both the male and the female groups (0.7 vs. 0.4 and 1.0 vs. 1.6).

The psychosocial factors at enrolment showed significant differences between male and female MTX users and MTX non-users in UCLA Loneliness mean score (36.2 vs. 32.8 and 39.0 vs. 35.0), CSFQ mean score (49.1 vs. 46.7 and 36.4 vs. 37.4) and BDI mean score (7.0 vs. 5.6 and 10.5 vs. 8.9).

Men in the MTX user group had a lower mean BMI than that of men with MTX non-use (26.8 vs. 28.1), and a difference was also present in the frequency of current smoking (46.2% vs. 58.8%) and alcohol consumption per week (40.4% vs. 52.9%). However, adjusted analyses showed no significant change in any parameters or scores comparing men using MTX with men not using MTX (Table 3, column E). The results must be considered in the light of the small sample size included in these analyses.

More women in the MTX-user group than those in the MTX non-user group had a CCI of 0 (64.6% vs 45.3%), indicating no comorbidities at the time of diagnosis. The adjusted analyses found a significant change in the UCLA Loneliness score between female MTX users and female MTX non-users (mean difference 4.6; CI 1.5–7.7) (Table 3, column F).

## Discussion

This study amongst RA patients investigated differences between MTX users and MTX non-users in selected PROMs and compared these between men and women. Participants were characterized by a low level of disability (mean HAQ score 0.8 at enrolment), a low comorbidity index, a low BDI and low scores of loneliness. In half of the participants, the CSFQ score indicated sexual dysfunction.

We found statistically significant gender differences in physical functioning, sexual dysfunction, depression and fatigue at the time of enrolment.

The results revealed a higher HAQ score for women than men, both at the time of diagnosis and at the time of study enrolment. Similar gender differences have been found in other studies, with higher scores reported by women in the HAQ, the fatigue score (VAS) and the PtGA score (VAS) [indicating a lower physical function], increased fatigue and a more negative disease impact in women than that in men [2, 43]. According to some studies, such gender differences might be explained by a tendency for female patients to evaluate symptoms more severely than male patients [4, 44], while men might tend to underestimate their symptoms [45]. The preponderance of female patients may result in outcome measures more likely being sensitive to women’s experiences. However, other explanations should be considered since clinical RA measurements may reflect biological differences (e.g., increased pain receptors in women), with a potentially more severe symptom picture in women [4, 46].

In line with other studies [24, 27], we found an increased Beck’s Depression Index (indicating more depressive symptoms) and a lower score in CSFQ (indicating more sexual dysfunction) in women with RA compared to men. A Danish study amongst the general population found a female predominance in depression, but differences were only significant concerning minor depression [47].

Gender differences in sexual dysfunction are present in the general population as well. For example, a Danish population study [48] found that 3.4% of partnered men vs 9.9% of partnered women reported hypoactive sexual desire disorder within the past 4 weeks.

The higher depression scores and low CSFQ scores in our study could add to the lowered well-being amongst female patients shown in some studies [27, 49]. However, other studies found no gender difference in the prevalence of depression amongst patients with RA [1, 50]. These differences may be due to scale measurement bias and would be worthy of further exploration using a male-specific depression scale that addresses ways through which men traditionally express depression (i.e., anger, aggression, substance abuse, self-sabotage and risk-taking behaviours) [51]. Again, this warrants future attention to the psychometric properties of the instruments used in clinical settings. Thus, clinicians should be aware that gender bias may be present when interpreting the scores of the HAQ or the BDI [43].

The multivariate analyses showed significant differences in self-reported loneliness between MTX users and MTX non-users. A relation between loneliness and MTX treatment (or other treatments for RA) has not previously been identified, while one study found living alone to be negatively associated with treatment adherence, whereas good mental health was positively associated with treatment adherence [52]. High loneliness scores may negatively impact medication taking and decrease adherence to treatment. In contrast, low loneliness scores could indicate more social support and help increase treatment adherence [23]. Since this sample’s overall loneliness scores were low, this must be considered when assessing the results.

The study found significant differences between women and men using MTX, with poorer outcomes for women in several parameters. A recent systematic literature review [53] analysed a large number of studies and several predictive models concerning MTX response within 3 to 6 months from the initial diagnosis, and it concluded that female gender, along with current smoking and/or rheuma-factor positivity, was a vague predictor of non-response to MTX. Almalag et al. [54] found female gender to be significantly associated with MTX intolerance and connected this difference to gender-specific pharmacokinetics. Female gender as a predictor of non-response/not using MTX aligns with our findings, although we did not test for RF positivity in the adjusted analyses.

In the adjusted analyses, smoking was found to be less likely in women than that in men––both in general and comparing male MTX users with female MTX users. This is in line with gender differences in smoking amongst the general Danish population [55]. Unlike one previous study [13], we did not observe any association between smoking and poor MTX use/response.

Our study reported significantly higher pain scores (VAS) in female MTX users than those in male MTX users. Results from other studies are contradictory regarding pain as a predictor of MTX response. One study found no impact on pain response [56], while an association of increased pain levels with a negative response to MTX therapy was reported in another study [54].

Comparisons between female and male MTX non-users showed increased disability measured by the HAQ score and increased sexual dysfunction for women not using MTX. These differences may reflect the general gender differences mentioned above since other studies, including ours, report increased sexual dysfunction in female RA patients(25) and increased HAQ scores in women compared to men [57]. Another explanation for the significant differences between women using MTX and women not using MTX (and female and male MTX users), along with the lack of differences between male MTX users and male MTX non-users, might be that the included PROMs lacked sensitivity to detect relevant changes in men.

The low number of male participants makes it difficult to reach firm conclusions on gender differences, and consequently, caution is needed in group comparisons. However, the study’s female-to-male ratio reflects the clinical population of RA patients (one male to four females).

Selection bias may have occurred, as participants were recruited after consultation in the outpatient department. This may have favoured participants with more disease activity/more severe arthritis. Conversely, patients declining to participate may have been experiencing more negative impacts of RA.

The data on MTX use could have been more detailed about reasons for MTX non-use, and data details were generally limited due to incomplete medical records. Further, the cross-sectional study design did not allow for exploring causal effects.

In conclusion, the results revealed better physical function at the time of diagnosis and a higher loneliness score in MTX users compared to MTX non-users in general. There were no significant differences in Beck’s Depression Index or the CSFQ score. However, women reported worse scores than men in several PROMs. Gender differences were also evident when comparing MTX users to MTX non-users, with female MTX non-users reporting worse PROMs than male MTX non-users. This may reflect that female RA patients are situated in more vulnerable positions than male RA patients. Still, it may also imply that the clinical measures used in rheumatology are more sensitive to adversities affecting women than men. Thus, gender differences should be considered when assessing patients’ symptoms and needs and when selecting or developing instruments to monitor various PROs.

## Data Availability

The datasets generated during and/or analysed during the current study are available from the corresponding author upon reasonable request. Part of the data was presented in a PhD thesis from SDU Denmark, and an abstract was submitted to the EULAR Conference. The latter was published in the EULAR abstract book, Annals of the Rheumatic Diseases Jun 2021, 80 (Suppl 1) 1468; 10.1136/annrheumdis-2021-eular.2039.
